# Adaptation of *Arabidopsis thaliana* to the Yangtze River basin

**DOI:** 10.1186/s13059-017-1378-9

**Published:** 2017-12-28

**Authors:** Yu-Pan Zou, Xing-Hui Hou, Qiong Wu, Jia-Fu Chen, Zi-Wen Li, Ting-Shen Han, Xiao-Min Niu, Li Yang, Yong-Chao Xu, Jie Zhang, Fu-Min Zhang, Dunyan Tan, Zhixi Tian, Hongya Gu, Ya-Long Guo

**Affiliations:** 1 State Key Laboratory of Systematic and Evolutionary Botany, Institute of Botany, Chinese Academy of Sciences, Beijing, 100093 China; 2 University of Chinese Academy of Sciences, Beijing, 100049 China; 3 Xinjiang Key Laboratory of Grassland Resources and Ecology, College of Grassland and Environment Sciences, Xinjiang Agricultural University, Urümqi, China; 4 State Key Laboratory of Plant Cell and Chromosome Engineering, Institute of Genetics and Developmental Biology, Chinese Academy of Sciences, Beijing, 100101 China; 5 State Key Laboratory for Protein and Plant Gene Research, College of Life Sciences, Peking University, Beijing, 100871 China; 6 The National Plant Gene Research Center, Beijing, 100101 China

**Keywords:** *Arabidopsis thaliana*, Population genomics, Adaptation, Yangtze River basin

## Abstract

**Background:**

Organisms need to adapt to keep pace with a changing environment. Examining recent range expansion aids our understanding of how organisms evolve to overcome environmental constraints. However, how organisms adapt to climate changes is a crucial biological question that is still largely unanswered. The plant *Arabidopsis thaliana* is an excellent system to study this fundamental question. Its origin is in the Iberian Peninsula and North Africa, but it has spread to the Far East, including the most south-eastern edge of its native habitats, the Yangtze River basin, where the climate is very different.

**Results:**

We sequenced 118 *A. thaliana* strains from the region surrounding the Yangtze River basin. We found that the Yangtze River basin population is a unique population and diverged about 61,409 years ago, with gene flows occurring at two different time points, followed by a population dispersion into the Yangtze River basin in the last few thousands of years. Positive selection analyses revealed that biological regulation processes, such as flowering time, immune and defense response processes could be correlated with the adaptation event. In particular, we found that the flowering time gene *SVP* has contributed to *A. thaliana* adaptation to the Yangtze River basin based on genetic mapping.

**Conclusions:**

*A. thaliana* adapted to the Yangtze River basin habitat by promoting the onset of flowering, a finding that sheds light on how a species can adapt to locales with very different climates.

**Electronic supplementary material:**

The online version of this article (doi:10.1186/s13059-017-1378-9) contains supplementary material, which is available to authorized users.

## Background

Global climate change has a profound influence on human health, food security, and biological diversity as it greatly taxes the ability of organisms to adapt to new environments [1–3]. A fundamental biological question that has recently emerged concerns how best to resolve the mismatch between organisms and human-altered environments. To avoid the tremendous cost of phenotype-environment mismatch, it is important to understand how organisms adapt to new habitats. The understanding of adaptation in constant environments, such as in serpentine soil using plants, or in experimental evolution using microorganisms, has progressed steadily [4, 5]. However, the mechanisms through which adaptation proceeds in heterogeneous natural environments are largely unknown. One of the major challenges in this area is that the genetic basis of adaptation to climate change is largely unknown.

Here, we use the plant model species *Arabidopsis thaliana* to address this fundamental question in the context of its adaptation in natural environments. *A. thaliana* is widely distributed across the temperate region in the northern hemisphere, including the Yangtze River basin, a region that is distant from its origin place of Europe/North Africa [6–9]. At several geographic scales in its native Eurasian range, *A. thaliana* demonstrates evidence of local adaptation [9–16]. Therefore, *A. thaliana* is a good model system to understand the mechanism of adaptation in natural environments at a global level [13, 16–19].

*A. thaliana* originated in Europe/North Africa [8, 9, 20, 21] and the Yangtze River basin is the most south-eastern edge of *A. thaliana*’s native habitats [22, 23]. The environment of the Yangtze River basin is tremendously different compared with both its origin in Europe/North Africa and other regions between the Yangtze River basin and Europe/North Africa where *A. thaliana* is found. Of the 19 climate variables (Additional file 1: Table S1), the temperature seasonality (bio4) and the annual precipitation (bio12) are the most differentiated climate variables among the different regions (Additional file 2: Figure S1). Therefore, it is of great interest to know how this species could adapt to the faraway south-eastern habitats with such distinct environments.

Selective sweep scans and quantitative genetics provide robust and efficient approaches to identify genetic variants correlated with adaptation [19, 24–26]. To understand how this model species could adapt to this region, we performed population genomics analyses and genetic mapping for flowering time variation, one of the most important life history traits correlated with fitness. We found that the Yangtze River *A. thaliana* population is unique and diverged 61,409 years ago from its ancestor population with two independent waves of gene flows afterwards; it expanded across the Yangtze River basin over thousands of years. Genes that correlated with biological regulation processes, such as flowering time, immune and defense response processes could have contributed to the adaptation of the Yangtze River population. Our results highlight how a plant species could adapt to a new climate.

## Results

### The Yangtze River population is unique

**Fig. 1:**
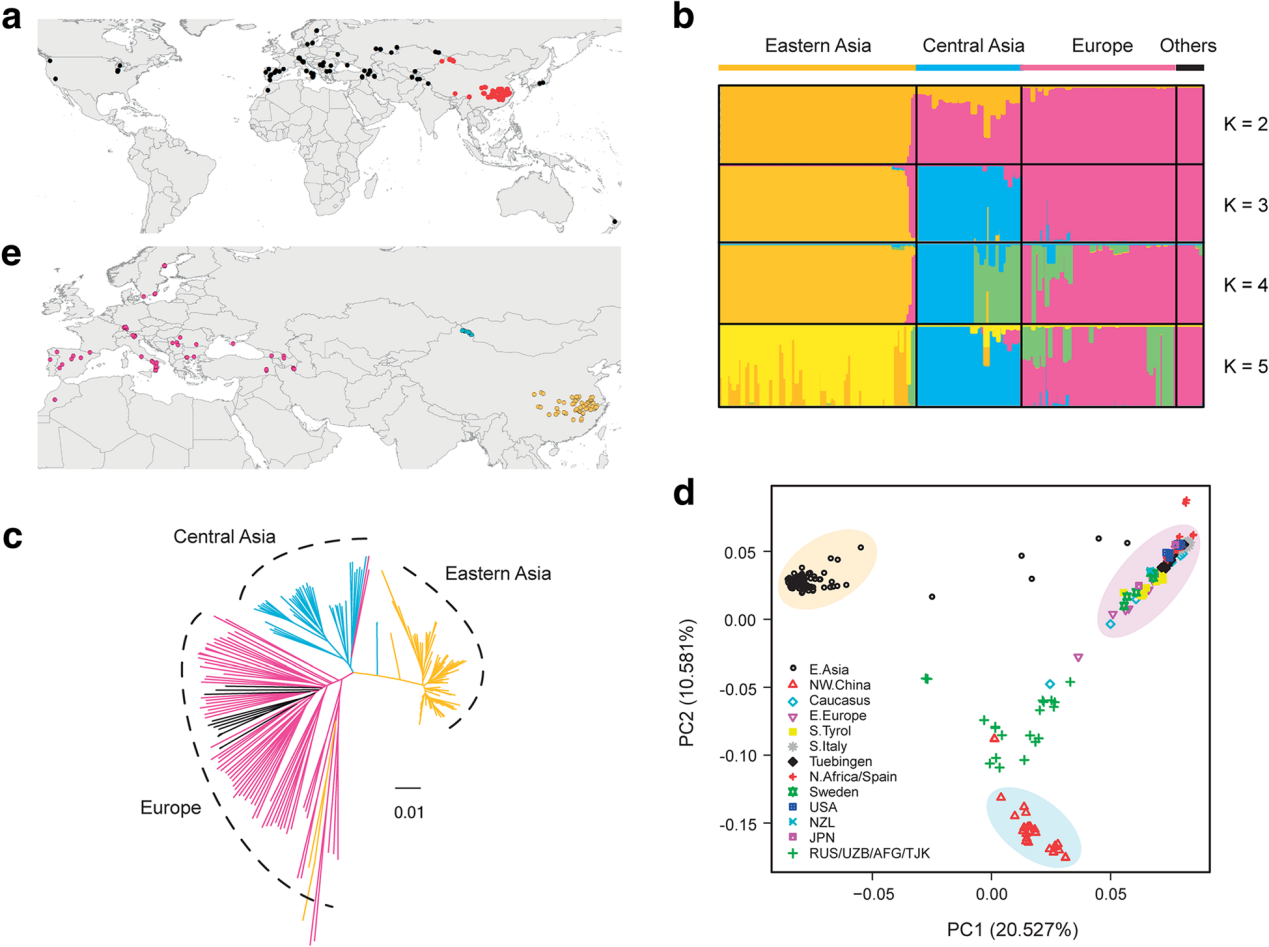
Geographic locations and population structure of *A. thaliana*. **a** *Map* of the locations sampled (*black points*) and sequenced (*red points*) in this study. **b** Population structure of *A. thaliana* based on admixture analysis of strains from (**a**). “Others” indicates strains from USA, Japan, and New Zealand, which most probably reflects recent introduction given that *A. thaliana* originated in Europe. **c** Phylogenetic tree of *A. thaliana* strains from (**a**). *Black lines* indicate strains from USA, Japan, and New Zealand. **d** Principle component analysis (PCA) of *A. thaliana. Shaded areas* indicate the populations from different regions: *pink* refers to the European population (popE), *blue* refers to popN, and *yellow* refers to popY. **e** The locations of different populations color-coded as in (**d**)

We sequenced 118 strains of *A. thaliana* across north-western China (mainly from the Altai Mountains) to south-eastern China along the Yangtze River (Fig. 1a and Additional file 3: Table S2). Each strain was sequenced to at least 18× coverage (average = 31.97×), which amounts to 3772.59 × coverage in total. From these genome sequences, we called 2.66 million single nucleotide polymorphisms (SNPs) and 0.58 million indels (Additional file 2: Figure S2), using the Col-0 strain as the reference genome. The SNPs called from the 118 strains sequenced in this study and SNPs extracted from 103 geographically representative genomes of the 1001 Genomes Project (Additional file 4: Table S3 for the detail) [10, 14, 27] were integrated together to represent the worldwide strains (Fig. 1a).

To explore the relationship among samples, admixture analysis, phylogenetic analysis, and principal component analysis (PCA) were conducted. These analyses suggested that these 221 strains, with some intermediate strains, could be divided into three major groups (eastern Asia, central Asia, and European/North Africa [hereafter referred to as Europe]), roughly consistent with their geographical origin (Fig. 1b–d). Phylogenetic analyses using two close relatives, *Arabidopsis lyrata* and *Capsella rubella*, as outgroups suggested that the Iberian Peninsula and North Africa strains are located at the basal position of the phylogenetic tree and confirmed that they are relicts [7, 9] (Additional file 2: Figure S3). A small number of strains from different geographical regions formed a clade, which most probably reflects relicts or recent introduction. For example, for those strains grouped with Europe/North Africa samples, three strains from south-western China (Tibet and Yunnan provinces) could be relicts, while strains from USA, Japan, and New Zealand that clustered with European sample could be recent introductions (Additional file 2: Figure S3). In the following analysis, we excluded the outlier strains that could disturb the local adaptation analysis, based on both phylogenetic and PCA results (Fig. 1d and Additional file 2: Figure S3). In this way, the final subsets included 86 strains from the Yangtze River basin (hereafter referred to as popY), 25 strains from north-western China (popN) to represent the central Asian population, and 67 strains from Europe/North Africa (popE) (Fig. 1d and e; Additional file 3: Table S2 and Additional file 4: Table S3). Simulation analyses suggested that the sample size we selected from the Yangtze River population is large enough to cover all the possible genetic variants (Additional file 2: Figure S4).

PopE has more SNPs, a total of 4,673,541, than either popY (*n* = 1,083,605) or popN (*n* = 975,715). PopE also has the highest number of private SNPs (*n* = 3,725,836) compared with popN (*n* = 273,787) and popY (*n* = 441,460). Furthermore, nucleotide diversity was highest in popE (*π* = 6.09 × 10^–3^), compared with popN (2.78 × 10^–3^) and popY (2.08 × 10^–3^) (Additional file 2: Figure S5). These results confirm that popE is the ancestral population [8, 9]. The *A. thaliana* samples that we studied make up three natural major groups, with popY from the Yangtze River basin being a uniform population.

### The Yangtze River population was recently established

**Fig. 2:**
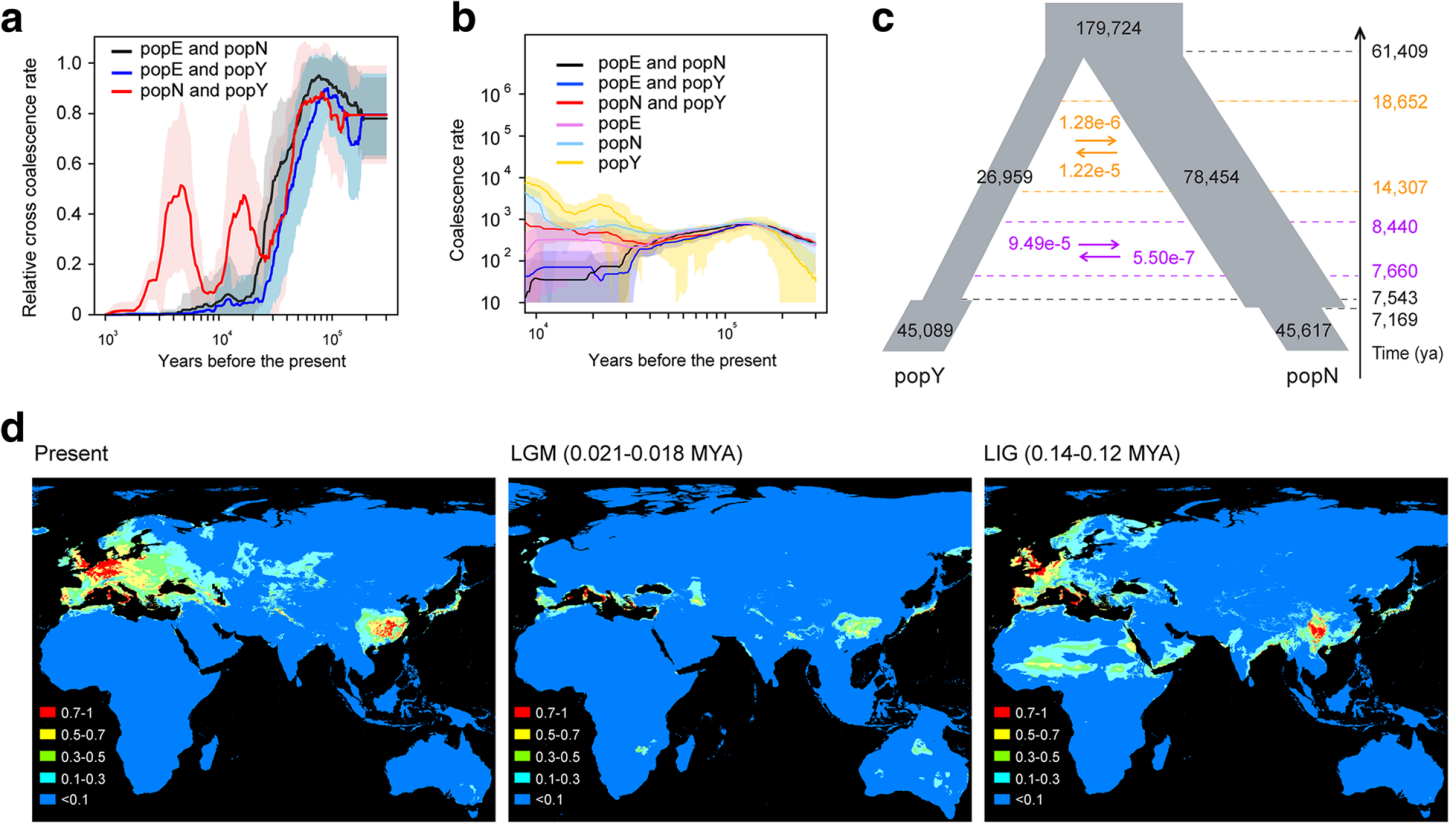
Demographic history of *A. thaliana*. **a** Relative cross coalescence rate reveals the age and pace of divergence between two populations. The two populations are well-mixed if the relative cross coalescence rate is 1 and fully separated when the value is 0. *Solid lines* represent means and *shading* represents standard deviations (50 random samplings). **b** Coalescence rates for pairs of individuals within and between populations. **c** The best demographic model of the two populations of *A. thaliana*. The width of the *boxes* represents the relative effective population size and *arrows* represent the migration between popN and popY. **d** Predicated distributions of *A. thaliana* based on ecological niche modeling. Areas in different *colors* indicate the various possibilities (0–1) of suitable habitats for *A. thaliana*. LGM last glacial maximum, LIG last interglacial, MYA million years ago

To clarify the genetic separation among populations of *A. thaliana*, we performed a multiple sequential Markovian coalescent (MSMC) analysis to estimate the relative cross coalescence rate [28]. By analyzing four haplotypes for each pair of populations, we found that all relative cross-coalescence rates between any two populations were similar and exhibited a gradual decline since the last glacial period (Fig. 2a). In contrast to the relative cross coalescence rates between popE and popN or popY, which completely diverged during the last glacial period, popN and popY diverged since then but with gene flow at two different periods, before separating completely about a few thousand years ago.

To reflect the historical processes for the different populations, we calculated the distribution of coalescence times as conducted in a previous study [9]. Coalescence rate is an indication of relatedness, with higher ones indicating closer relationship and smaller population sizes. From the analysis of two haplotypes, the results suggested that, since the last glaciation, coalescence rates within popN and popY were much higher than that for popE; and coalescence rates between members of popN and popY were higher than those between popE and popN or popY (Fig. 2b).

Furthermore, we employed fastsimcoal2 [29] to infer the demographic history of the *A. thaliana* popN and popY populations, combining the findings with those of the aforementioned MSMC study. Four alternative models with different extents of gene flow and varying population sizes were investigated (Additional file 2: Figure S6). The best fit model had two waves of asymmetrical gene flow, which is consistent with the gene flow at two different periods in the MSMC analysis (Fig. 2a). Under the best model, popN and popY diverged 61,409 years ago from an ancient population of size 179,724 into sizes of 26,959 and 78,454, respectively (Fig. 2c, see Additional file 1: Table S4 for the detail). Gene flow existed at two time stages, between 18,652 and 14,307 years ago, and between 8440 and 7660 years ago, although both of these gene flow events were weak. Following that, since 7543 years ago, popY exhibited a notable expansion and reached the size of 45,089, and distributed across the Yangtze River basin, while popN went through a reduction to 45,617, about 7169 years ago.

Ecological niche modeling (ENM) based on the *A. thaliana* distribution information (Additional file 5: Table S5) indicates that there were widely suitable habitats, roughly connected between the Yangtze River basin and the southern slopes of the Himalayas Mountains around the last interglacial period (Fig. 2d). This result revealed that the extant *A. thaliana* population of the Yangtze River basin could be derived from the eastward dispersion via the Himalayas, in agreement with previous proposals [22]. This observation is also supported by the phylogenetic results, in which samples from central Asia (including popN) are the most closely related lineage of popY (Additional file 2: Figure S3). In summary, we found that glacial cycle is one of the major determinants of the demographic history of *A. thaliana*. PopY diverged about 61,409 years ago from its ancestor and expanded across the Yangtze River basin thousands of years ago.

### Pervasive selection and genomic signatures of local adaptation of the Yangtze River population

**Fig. 3:**
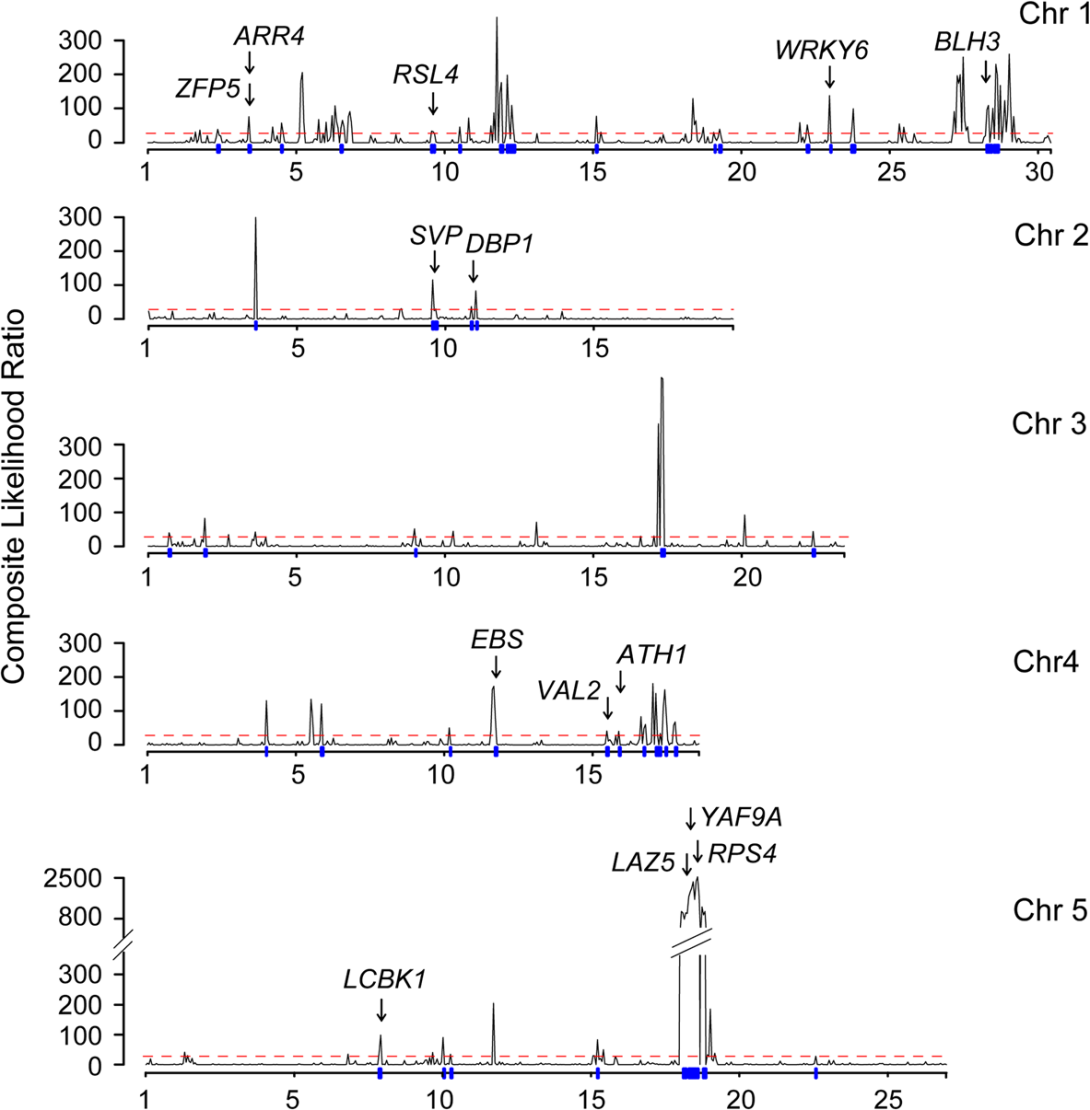
Positive selection analysis in the Yangtze River basin population. *Dashed red line* indicates the cut-off of composite likelihood ratio and *vertical blue lines* across the *x-axis* indicate the overlapped regions that are under positive selection in both SweepFinder2 and OmegaPlus

Abrupt geographical change in allele frequency is evidence of strong local adaptation [9]. To detect genes that are under positive selection and are important for adaptation, we searched the genomes for a selective sweep signal using a site frequency spectra (SFS)-based method (SweepFinder2) (Fig. 3) and a linkage disequilibrium (LD)-based method (OmegaPlus) (Additional file 2: Figure S7). The overlapped regions under selection between the two methods were regarded as the candidate regions of selection. In total, there were 530 protein-coding genes under positive selection (Fig. 3, see Additional file 6: Table S6 for the detail). These genes might have contributed to the adaptation of popY to the Yangtze River basin.

Gene Ontology (GO) analysis of the candidates under positive selection detected five significantly enriched biological process GO terms including immune response, innate immune response, immune system process, defense response, and biological regulation (false discovery rate [FDR] < 0.01; Additional file 2: Figure S8). The biological regulation processes comprised diverse genes, such as multiple gene candidates related to flowering (*SVP*, *DBP1*, *YAF9A*, *BLH3*, *VAL2*, *EBS*, *ATH1*) [30–37], response to temperature stress (*LCBK1*) [38], root hair development (*ZFP5*, *RSL4*, *WRKY6*) [39–41], and circadian period (*ARR4*) [42]. For the immune response genes, 19 genes were enriched in all of the four GO terms at the same time except the biological regulation GO term, of which nine are nucleotide-binding, leucine rich repeat (NB-LRR) genes, including the well-known genes *RPS4* and *LAZ5*. RPS4 interacts with another NB-LRR protein RRS1-R and triggers defense response [43, 44]. *LAZ5* encodes a TIR-class NB-LRR gene and could activate cell death [45, 46]. Overall, the selection scan suggested that genes enriched in biological regulation processes, such as flowering time, immune response, and defense response, could play an important role during the establishment of the Yangtze River population.

### Genomic regions associated with flowering time variation

**Fig. 4:**
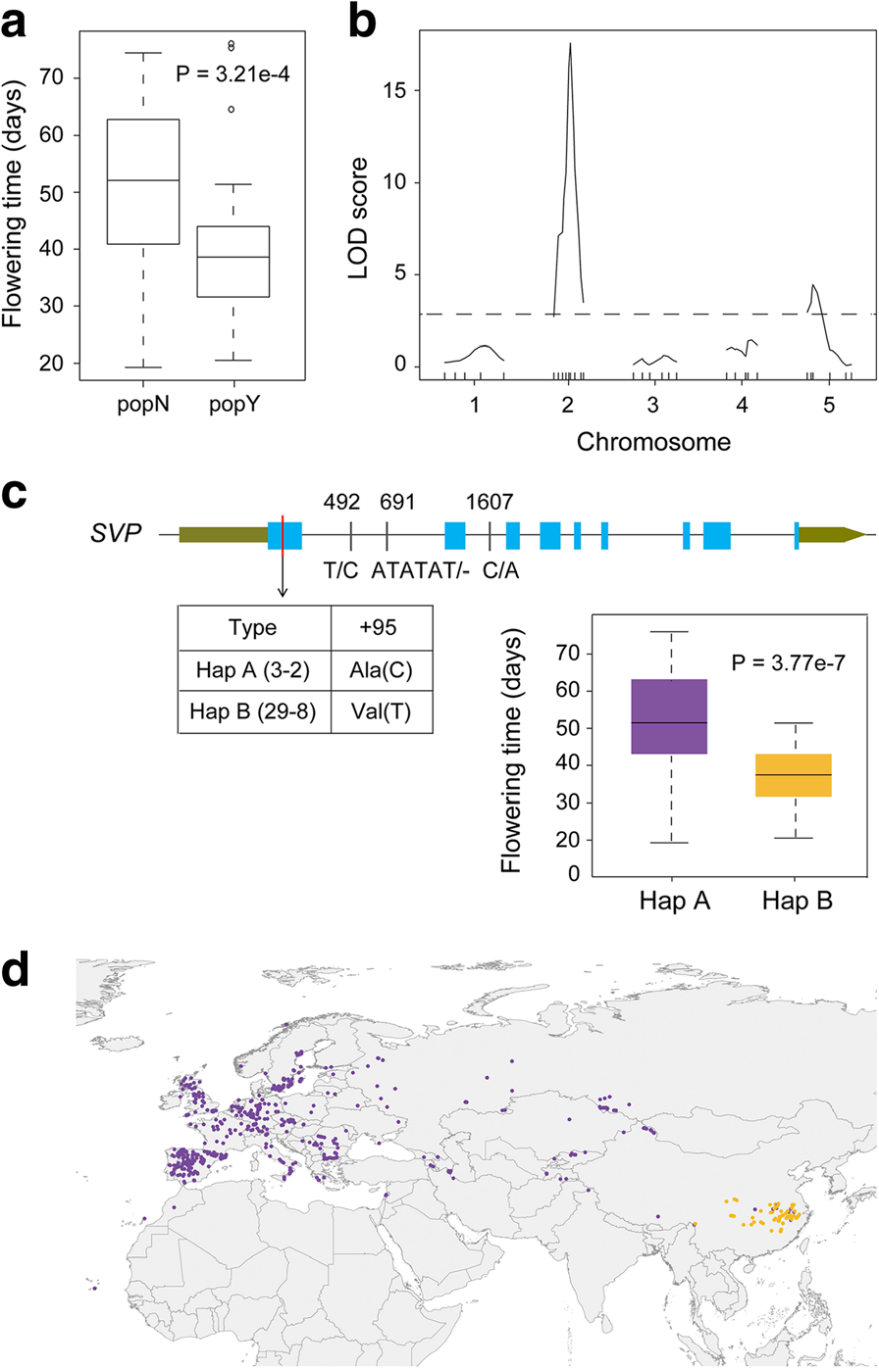
Genomic regions associated with flowering time variation. **a** Flowering time variation between popY and popN. **b** QTL mapping analysis of flowering time between 3-2 and 29-8. The *dashed horizontal line* indicates the LOD threshold for QTLs (LOD = 2.9). **c** Sequence variation of *SVP* between 3-2 and 29-8; association between haplotypes and flowering time among 98 accessions. **d** Distribution of haplotypes across the world

Given that some outlier loci from genome-wide selection scans might not be actually adaptive [47] and adaptation to the new climate could involve different traits [1], association between fitness related traits and genomic variation is a robust way to validate genes that are found by selection scans [48, 49]. Flowering time is an important fitness trait and there was huge flowering time variation within or between popY and popN (Additional file 3: Table S2). In particular, popY is significantly early flowering than popN (Fig. 4a).

To clarify the genetic basis of flowering time variation, we constructed F_2_ population (1158 plants in total) using two extreme accessions with contrasting flowering time (3-2 flowered after 50.33 days and 29-8 after 24.87 days), and identified *SVP* as the causal locus (Fig. 4b). To identify the causal gene, 86 plants of F_2_ individuals were used in the analysis. Quantitative trait locus (QTL) mapping identified two QTLs on chromosomes 2 and 5 that were responsible for the flowering time variation and the locus on chromosome 2 explained a larger fraction of the flowering time variation compared with that on chromosome 5 (60.9% vs 21.6%; Fig. 4b). To fine-map the locus on chromosome 2, we analyzed 184 early-flowering F_2_ plants and narrowed the candidate region to 130 kb (Additional file 1: Table S7). Within this region between the two accessions, there are only four polymorphisms in four different genes that induced amino acid changes, which are assumed to be functionally important [50]. Only one of these four genes, *SHORT VEGETATIVE PHASE* (*SVP*, AT2G22540) is a well-known negative regulator of the onset of flowering that could be degraded at high temperature and promote flowering [51, 52]. We divided the 98 accessions of popN and popY with the flowering time data, into two different haplotypes according to the non-synonymous polymorphism. There is significant difference in the flowering time between the two haplotypes (Fig. 4c).

The non-synonymous polymorphism between the two haplotypes leads to one amino acid substitution (Ala^32^/Val^32^) in exon1 located in the MADS-box domain, which has been demonstrated to generate a loss-of-function (LOF) allele and could promote flowering [34] (Fig. 4c). Within the 881 genomes from the 1001 Genomes Project and the 118 genomes sequenced in this study (see Additional file 7: Table S8 for the details), we found that the amino acid substitution (Ala^32^/Val^32^) only existed in the Yangtze River region and was almost fixed, consistent with the scenario of positive selection on the *SVP* gene (Fig. 4d). However, this mutation has been identified in the natural accessions of Pakistan and Japan [34] that are not included in the present study. We concluded that the amino acid mutation of the *SVP* gene should have contributed to the adaptation to the Yangtze River basin.

## Discussion

Global climate change has had a tremendous impact on the fitness of various organisms, mainly due to the lagging adaptation to climate change [53]. Understanding the adaptation of plants to new environments is a robust and practical way to understand the mechanisms behind this mismatch [3, 54]. In particular, it is largely unknown which kind of molecular processes or mechanisms are the determinant factors during adaptation process. To fully clarify the complete picture of local adaptation is challenging and complicated, as the process involves different factors, including identifying the genomic loci under selection, the phenotypes that selection is acting upon, and the external conditions driving the selection [55]. The classic scan of genes under positive selection and the mapping of genes correlated with the adaptive traits, such as flowering time, are robust ways to identify genes correlated with adaptation [9, 55].

The present study revealed the demographic history of *A. thaliana* at the global level of its natural habitats and indicates that the Yangtze River population is a unique population that diverged 61,409 years ago and expanded recently to the Yangtze River basin. This knowledge is a great opportunity to address how plants adapt to the diverse habitats in natural environments. We found that biological regulation processes, such as flowering time, immune and defense response processes could be important in this adaptation process. Particularly, *SVP* LOF mutation has been under positive selection and is nearly fixed in the Yangtze River population. Given that *SVP* is an important gene to allow plants to respond to ambient temperature changes in the context of global climate change [56], it must play an important role in the adaptation of the plant to the Yangtze River basin, the most south-eastern of *A. thaliana*’s native habitats. Consistently, during the range expansion of an invasive plant *Lythrum salicaria*, earlier flowering is important for the adaptation [54]. Many more studies are necessary to reveal the genetic basis of adaptation; for example, further analyses of the genes under positive selection in this study will be insightful for understanding the genetic basis of adaptation, mapping another QTL on chromosomes 5, and characterizing the mechanism behind the flowering time variation between the two accessions (3-2 and 29-8). In addition, given that we found that there are gene flows between popN and popY at two different periods (Fig. 2a), it would be intriguing to know to what extent these gene flows have contributed to adaptation. Overall, this study greatly progresses our understanding of the adaptation in plants by exploring the genetic variations and adaptation of the worldwide samples of *A. thaliana*.

## Conclusions

Adaptation is a robust way to deal with the challenge of global climate change. Examining recent range expansion aids our understanding of how organisms evolve to overcome environmental constraints. Our results suggest that *A. thaliana* dispersed thousands of years ago to the Yangtze River basin, the most south-eastern edge of its native habitats. In addition, we demonstrate that flowering time variation related genes and immune response genes, particularly *SVP*, have contributed to the adaptation to the Yangtze River basin. This study highlights the importance of adaptation and demonstrates the genetic basis of adaptation in plants.

## Methods

### Plant materials and resequencing

A total of 118 strains were collected from north-western China and south-western China along the Yangtze River basin to eastern China [57] (Additional file 3: Table S2). Genomic DNA was extracted from the seedlings using the CTAB method [58]. Paired-end sequencing libraries with insert size around 500 bp were constructed. One hundred base-pair paired-end reads were sequenced using Illumina HiSeq 2000 for 91 samples and 150 bp paired-end reads were sequenced using Illumina HiSeq X Ten for the other 27 samples. For flowering time measurements, at least 11 plants were sowed for each strain in the greenhouse at 20 °C and 40–65% humidity with a 16-h photoperiod. Flowering time was assayed as the day of the first flower anthesis and the average of flowering time from each strain was regarded as flowering time [59].

### Identification of SNPs and indels

Paired-end reads were mapped to the TAIR10 reference genome (www.arabidopsis.org) using Burrows–Wheeler Alignment tool (version 0.6.2) [60], allowing up to 4% mismatches and one gap. Next, the rmdup function of Samtools (version 0.1.8) [61] was used to remove reads that were duplicated in library preparation or sequencing. Finally, reads were locally realigned with the Genome Analysis Toolkit (GATK version 2.1.8) [62] Indel Realignment tool that performs realignment around indels to avoid alignment errors. SNPs and indels were called using the UnifiedGenotyper tool packaged in GATK with default parameters. Extra filtration steps were applied to the raw SNPs and indels using the built-in function VariantFiltration, including quality (Q) ≥ 30, mapping quality (MQ) ≥ 20, quality-by-depth ratio (QD) ≥ 10, ReadPosRankSum ≥ − 8.0, depth coverage (DP) ≥ 3, probability of strand bias (FS) ≤ 10.0 (FS ≤ 200.0 for indels), and no more than three SNPs within 10 bp.

### Population genetics analysis

Besides the 118 strains sequenced in this study, 103 published strains were included for analysis [10, 14, 27] (Additional file 4: Table S3) and thus 221 strains in total were used in the study. The biallelic SNPs with information in at least 219 strains (in total, 1.97 million SNPs) were used to perform the population genetics analyses. ADMIXTURE [63] was used to estimate the genetic ancestry of each sample, specifying a range of 2–5 hypothetical ancestral populations. PCA was performed with EIGENSOFT (version 4.2) [64]. The unrooted neighbor-joining tree was constructed with PHYLIP (version 3.695) [65]. In addition, a neighbor-joining tree using the third codon site of 16,047 orthologous genes across the three closely related species, *A. thaliana* (221 strains), *Arabidopsis lyrata* (MN47) [66], and *Capsella rubella* (MTE) [67], was constructed, with MN47 and MTE as the outgroups. Orthologous genes among *A. thaliana*, *A. lyrata*, and *C. rubella* were identified by InParanoid [68] with default parameters. Nucleotide diversity *π*, Watterson’s estimator θ, and *F*_ST_ were calculated in a 200-kb sliding window with a step size of 10 kb.

### Demographic and ecological niche analyses

The demographic history of *A. thaliana* was inferred using the MSMC model [28] based on two or four haploid genomes with default parameters. As the *A. thaliana* plant self-fertilizes, the genome of each strain can be considered as a haplotype sequence when heterozygous sites are excluded. Only homozygous SNP sites without missing data were used in the analysis. For two haplotypes, two strains were randomly extracted from the same population (popE, popN, or popY) or two populations (one haplotype from each population). For four haplotypes, four strains were randomly extracted from the same population or two different populations (two haplotypes from each population). In each analysis, 50 rounds of random samplings were performed to estimate the mean and standard deviation of the relative cross coalescence rate or the coalescence rates along the evolutionary time.

Fastsimcoal2 [29] was used to infer the demographic parameters of popY and popN. First, the site frequency spectra (SFS) was computed for the 399,165 non-coding SNPs that have no missing site in any of the samples. Four alternative models with different extents of gene flow and varying population sizes were compared, using Akaike’s information criterion (AIC) and Akaike’s weight of evidence [29]. The timespans of the gene flow were set according to the observations in Fig. 2a and effective population sizes were set according to the results of Fig. 2b. The best parameter estimates under each model were obtained from 50 independent runs with a minimum of 100,000 and a maximum of 1,000,000 coalescent simulations as well as 10–40 cycles of the likelihood maximization algorithm. SFS entries with support from < 10 SNPs were ignored [29]. The 95% confidence intervals for each parameter were computed based on 100 parametric bootstrapping datasets simulated according to the estimations under the best model, using fastsimcoal2 again. In this study, the generation time (g) was set as one year and the mutation rate was considered to be 7 × 10^–9^ per base per generation [69] and the recombination rate as 3.6 cM/Mb [70].

To reconstruct the potential distribution pattern of *A. thaliana* worldwide, ENM analysis was employed to predict the distribution of *A. thaliana* during three periods, including the present time, the time of last glacial maximum (LGM; 0.021–0.018 MYA) and the time of last interglacial (LIG; 0.14–0.12 MYA). In total, 291 geo-referenced and non-overlapped occurrence records of *A. thaliana* from our own field works and published articles [9, 10, 14, 27] were used; these records covered nearly the whole native ranges of *A. thaliana* in the world (Additional file 5: Table S5). The 19 environmental variables of the three periods used to perform ENM analysis were downloaded from the WORLDCLIM database (www.worldclim.org). Since the existence of strongly related environmental variables may over-fit models during ENM analysis, environmental variables were filtered so that no two variables had a pairwise Pearson correlation coefficient *r* > 0.7 or < – 0.7 (Additional file 1: Table S9). As a result, 11 environmental variables were used for the subsequent analysis (Additional file 1: Table S10). Ecological niche models were constructed using the present variables and projected for the other two historical variable datasets via maximum entropy in Maxent 3.3.3 [71] with default settings as in our previous study [72]. To identify the most significant climate variable that contributes to the distribution of *A. thaliana*, we performed PCA on the 19 environmental variables using R (www.r-project.org).

### Selection test and functional annotation

SweepFinder2 is an effective program that implements a powerful likelihood-based method for detecting recent positive selection or selective sweeps. SweepFinder2 is the first method that accounts for the effects of negative selection on diversity when searching for adaptive alleles [73]. SweepFinder2 scanned for positive selection in the folded site frequency spectrum (fSFS) for popY. The parameter -g was set to 50,000. In total, 10,000 1-Mb simulation datasets were generated as the null datasets based on the demographic parameters from the best model using Fastsimcoal2. The 10,000 simulation datasets were used to calculate a statistical cut-off with the same parameters as for the real data, allowing for a false-positive rate of 0.01%. After filtering with the threshold of 27.85, the neighbor sweep targets were merged to sweep regions.

To increase the ability to detect selective sweeps, OmegaPlus (version 2.3.0; a LD-based method) was used [74]. The ω statistic was computed at 10 kb intervals. The minwin and maxwin parameters were set to 10 kb and 100 kb, respectively. As in the Fastsimcoal2 method, 10,000 simulation datasets were used to calculate a statistical cut-off. The sweep targets adjacent to each other were subsequently merged to sweep regions after filtering with the cut-off (ω > 11.92). The overlap regions of the two methods were computed and those regions were regarded as the confident selective sweep regions. Genes within these regions were regarded as genes under selection. The software Cytoscape with the BiNGO plugin was used for GO analysis [75].

### QTL mapping

For QTL mapping of flowering time variation, 1158 individuals of F_2_ plants generated from 3-2 (female) and 29-8 (male) were used. Markers were identified based on the resequencing data, in which indel and SNP markers were called with Pindel (version 0.2.5a3) and GATK (version 2.1.8), respectively [76]. The genotype information of 32 markers across the whole genome, with an average density of 3.75 Mb/marker (Additional file 1: Table S11) and the flowering time of 86 F_2_ individuals, were used to perform QTL analysis using the R/qtl package with default parameters implemented in R (http://www.R-project.org).

### Statistical analysis

Statistical analyses were performed in R (www.r-project.org).

## Additional files

Additional file 1: Table S1. Environmental variables used in the ecological analysis. **Table S4.** Demographic parameters results from fastsimcoal2. **Table S7.** Fine mapping of causal locus on chromosome 2 with 184 early flowering plants from the F_2_ population of 3-2 × 29-8. **Table S9.** Significant Pearson’s correlation (*r* > 0.7 or < – 0.7) between bioclimatic variables in the distribution of all samples (outlined by green), with the retained variables during the ecological niche modeling analysis in red. **Table S10.** The contributions of the 11 bioclimatic variables (abbreviation in parentheses) to the Maxent models during ENM analysis for total and geographic populations, respectively, with their permutation importance index indicated in parentheses. Values corresponding to the three most significant variables are in red. **Table S11.** Markers used in the QTL mapping analysis. (DOCX 54 kb)

Additional file 2: Figure S1. PCA analysis of ecological differentiation among strains of *A. thaliana* based on 19 environmental variables using two discriminant principal components (PC) based on 291 geo-referenced and non-overlapped occurrence records of *A. thaliana* (Additional file 5: Table S5). Bio4 and bio12 are the most significant factors differentiating PC2 and PC1, respectively. All the pairwise comparisons of bio4 or bio12 among the three regions are significant (*P* < 0.001). **Figure S2.** Sequence variation of the 118 strains sequenced in this study. Intergenic represents intergenic region; gene represents genic region from ATG to TGA/TAA/TAG, including introns; 5’ UTR represents the untranslated region before start codon ATG; CDS represents coding sequence region; 3’ UTR refers the untranslated region after stop codon (TGA/TAA/TAG); total represents whole genome in total. **Figure S3.** Phylogenetic tree of all the 221 *A. thaliana* strains with outgroups. Numbers nearby a branch indicate the bootstrap value > 50% with 100 replicates. Strains from different regions were color-coded, pink: European strains, blue: central Asia strains, yellow: eastern Asia strains, green: others indicate strains from USA, Japan, and New Zealand, most probably reflects recent introduction, given *A. thaliana* originated in Europe. **Figure S4.** Saturation analysis of the 30 times random samplings of the Yangtze River population (popY) based on the recovery of the number of total SNPs. **Figure S5.** Genetic variation among different populations. **Figure S6.** Different models of demographic history between the two populations of *A. thaliana*. Model 4 is the best fit model; see Table S4 for the detailed demographic parameters for each model. **Figure S7.** Selection scans of genes under positive selection based on LD-based method (OmegaPlus). The dashed red line indicates the threshold of 0.01% based on simulation data sets. **Figure S8.** Over-representation (FDR < 0.01) of GO annotation categories in gene sets under selection. (DOCX 2316 kb)

Additional file 3: Table S2. Samples sequenced in this study. (XLSX 21 kb)

Additional file 4: Table S3. Previously sequenced samples that are used in this study. (XLSX 16 kb)

Additional file 5: Table S5. Geographic information of the representative samples used in the ecological niche modeling (ENM) analysis. The geographic region was divided via longitude range and latitude range: Europe (192 samples) = E8.54 ~ 38.28 and N31.47 ~ 61.36; central Asia (48 samples) = E42.22 ~ 90.34 and N37.29 ~ 58.01; eastern Asia (51 samples) = E98.63 ~ 120.37 and N26.75 ~ 33.15. (XLSX 21 kb)

Additional file 6: Table S6. Genes under positive selection based on the overlapping of the two different methods. (XLSX 14 kb)

Additional file 7: Table S8. The 999 accessions used for the *SVP* gene haplotype analysis. (XLSX 51 kb)

## Abbreviations

ENM: Ecological niche modelling
GO: Gene Ontology
MSMC: Multiple sequential Markovian coalescent
PCA: Principal component analysis
popE: Europe/North Africa population
popN: North-western China population
popY: Yangtze River basin population
QTL: Quantitative trait locus
