# Supplementary material for: Adaptation of Arabidopsis thaliana to the Yangtze River basin
Source: Genome Biol. 2017 Dec 28;18:239. doi: 10.1186/s13059-017-1378-9 (PMC5745794; doi:10.1186/s13059-017-1378-9)
Supplement: Supplementary file 1 — Environmental variables used in the ecological analysis. Table S4. Demographic parameters results from fastsimcoal2. Table S7. Fine mapping of causal locus on chromosome 2 with 184 early flowering plants from the F2 population of 3-2 × 29-8. Table S9. Significant Pearson’s correlation (r > 0.7 or < – 0.7) between bioclimatic variables in the distribution of all samples (outlined by green), with the retained variables during the ecological niche modeling analysis in red. Table S10. The contributions of the 11 bioclimatic variables (abbreviation in parentheses) to the Maxent models during ENM analysis for total and geographic populations, respectively, with their permutation importance index indicated in parentheses. Values corresponding to the three most significant variables are in red. Table S11. Markers used in the QTL mapping analysis. (DOCX 54 kb) [file 13059_2017_1378_MOESM1_ESM.docx]

**Table S1. Environmental variables used in the ecological analysis.**

| **Environmental variable** | **Annotation** | **Source** |
| --- | --- | --- |
| bio1 | Annual Mean Temperature | WORLDCLIM (www.worldclim.org) |
| bio2 | Mean Diurnal Range (Mean of monthly (max temp - min temp)) |  |
| bio3 | Isothermality (BIO2/BIO7) (* 100) |  |
| bio4 | Temperature Seasonality (standard deviation *100) |  |
| bio5 | Max Temperature of Warmest Month |  |
| bio6 | Min Temperature of Coldest Month |  |
| bio7 | Temperature Annual Range (BIO5-BIO6) |  |
| bio8 | Mean Temperature of Wettest Quarter |  |
| bio9 | Mean Temperature of Driest Quarter |  |
| bio10 | Mean Temperature of Warmest Quarter |  |
| bio11 | Mean Temperature of Coldest Quarter |  |
| bio12 | Annual Precipitation |  |
| bio13 | Precipitation of Wettest Month |  |
| bio14 | Precipitation of Driest Month |  |
| bio15 | Precipitation Seasonality (Coefficient of Variation) |  |
| bio16 | Precipitation of Wettest Quarter |  |
| bio17 | Precipitation of Driest Quarter |  |
| bio18 | Precipitation of Warmest Quarter |  |
| bio19 | Precipitation of Coldest Quarter |  |

**Table S4. Demographic parameters results from fastsimcoal2.**

| **Parameter** | **No gene flow** | **Continuing gene flow** | **One time gene flow** | **Two times gene flow** | | |
| --- | --- | --- | --- | --- | --- | --- |
|  |  |  |  | **Point estimates** | **95% Confidence intervals** | |
|  |  |  |  |  | **Lower limit** | **Upper Limit** |
| NACN | 199118 | 190718 | 204938 | 179724 | 172380 | 191131 |
| NN | 90027 | 43721 | 92840 | 45617 | 43731 | 79670 |
| NY | 48102 | 41029 | 48277 | 45089 | 43015 | 49482 |
| NAN | 37396 | 81687 | 55808 | 78454 | 60429 | 87010 |
| NAY | 15463 | 27343 | 16629 | 26959 | 23180 | 29565 |
| MIG2YN | - | 2.28E-06 | 3.36E-05 | 9.49E-05 | 1.84E-05 | 8.48E-04 |
| MIG2NY | - | 1.00E-06 | 5.51E-06 | 5.50E-07 | 1.04E-07 | 1.41E-04 |
| MIG1YN | - | - | - | 1.28E-06 | 6.80E-08 | 6.43E-06 |
| MIG1NY | - | - | - | 1.22E-05 | 8.90E-07 | 4.80E-05 |
| TMIG2Start | - | - | 7351 | 7660 | 5273 | 9553 |
| TMIG2End | - | - | 16327 | 8440 | 7312 | 9873 |
| TMIG1Start | - | - | - | 14307 | 10713 | 17444 |
| TMIG1End | - | - | - | 18652 | 14307 | 19649 |
| TDIV | 24381 | 58696 | 40933 | 61409 | 46031 | 66732 |
| TN | 1559 | 9619 | 763 | 7169 | 406 | 8789 |
| TY | 9465 | 9554 | 9284 | 7543 | 4441 | 9350 |
| Max EstLhood | -1823201.01 | -1815817.27 | -1814111.22 | -1814098.50 | - | - |
| AIC | 8396166.92 | 8362167.57 | 8354314.92 | 8354264.31 | - | - |
| ΔAIC | 41902.61 | 7903.27 | 50.61 | 0 | - | - |
| _w_ | 0 | 0 | 1.02E-11 | 1 | - | - |

NACN represents ancestral population size.

NN represents the population size of popN.

NY represents the population size of popY.

NAN represents ancestral population size of popN.

NAY represents ancestral population size of popY.

MIG1YN represents migration rate from popY to popN per generation of the first gene flow.

MIG1NY represents migration rate from popN to popY per generation of the first gene flow.

MIG2YN represents migration rate from popY to popN per generation of the second gene flow.

MIG2NY represents migration rate from popN to popY per generation of the second gene flow.

TMIG1Start represents the start time of the first migration.

TMIG1End represents the end time of the first migration.

TMIG2Start represents the start time of the second migration.

TMIG2End represents the end time of the second migration.

TDIV represents the divergence time of popN and popY.

TN represents the time of population size change in popN.

TY represents the time of population size change in popY.

Max EstLhood: log-likelihood of the best estimate.

AIC represents Akaike’s information criterion, AIC = 2d - 2ln(Lhood), where d is the number of parameters.

ΔAIC = AIC - min(AIC).

w represents Akaike’s weight of evidence.

**Table S7. Fine mapping of causal locus on chromosome 2 with 184 early flowering plants from the F_2_ population of 3-2 ×29-8.**

| **Marker location on chromosome 2** | **Genotype** | | | | | **29-8 frequency** | |  |
| --- | --- | --- | --- | --- | --- | --- | --- | --- |
|  | **29-8** | **Heterozygote** | | | **3-2** |  |  |  |
| 7416218 - 7417920 | 132 | | 46 | 6 | | | 0.842391304 | |
| 8800604 - 8800976 | 147 | | 32 | 5 | | | 0.885869565 | |
| 9295265 - 9295912 | 151 | | 28 | 5 | | | 0.896739130 | |
| 9502404 - 9503558 | 152 | | 27 | 5 | | | 0.899456522 | |
| **9547395 - 9548286** | **155** | | **24** | **5** | | | **0.907608696** | |
| **9580153 - 9580865** | **157** | | **22** | **5** | | | **0.913043478** | |
| **9615467 - 9616302** | **157** | | **22** | **5** | | | **0.913043478** | |
| **9648092 - 9648867** | **157** | | **22** | **5** | | | **0.913043478** | |
| **9678029 - 9678751** | **156** | | **23** | **5** | | | **0.910326087** | |
| 9726351 - 9726998 | 156 | | 23 | 5 | | | 0.910326087 | |
| 10011660 - 10012093 | 156 | | 23 | 5 | | | 0.910326087 | |
| 10537897 - 10538400 | 149 | | 30 | 5 | | | 0.891304348 | |
| 11818064 - 11818396 | 136 | | 42 | 6 | | | 0.853260870 | |

Markers in bold indicate the causal region.

**Table S9. Significant Pearson’s correlation (*r* > 0.7 or < -0.7) between bioclimatic variables in the distribution of all samples (outlined by green), with the retained variables during the ecological niche modeling analysis in red.**

|  | bio1 | bio2 | bio3 | bio4 | bio5 | bio6 | bio7 | bio8 | bio9 | bio10 | bio11 | bio12 | bio13 | bio14 | bio15 | bio16 | bio17 | bio18 |
| --- | --- | --- | --- | --- | --- | --- | --- | --- | --- | --- | --- | --- | --- | --- | --- | --- | --- | --- |
| bio1 |  |  |  |  |  |  |  |  |  |  |  |  |  |  |  |  |  |  |
| **bio2** |  |  |  |  |  |  |  |  |  |  |  |  |  |  |  |  |  |  |
| **bio3** |  |  |  |  |  |  |  |  |  |  |  |  |  |  |  |  |  |  |
| bio4 |  |  |  |  |  |  |  |  |  |  |  |  |  |  |  |  |  |  |
| **bio5** |  |  |  |  |  |  |  |  |  |  |  |  |  |  |  |  |  |  |
| bio6 |  |  |  |  |  |  |  |  |  |  |  |  |  |  |  |  |  |  |
| **bio7** |  |  |  |  |  |  |  |  |  |  |  |  |  |  |  |  |  |  |
| **bio8** |  |  |  |  |  |  |  |  |  |  |  |  |  |  |  |  |  |  |
| **bio9** |  |  |  |  |  |  |  |  |  |  |  |  |  |  |  |  |  |  |
| bio10 |  |  |  |  |  |  |  |  |  |  |  |  |  |  |  |  |  |  |
| bio11 |  |  |  |  |  |  |  |  |  |  |  |  |  |  |  |  |  |  |
| **bio12** |  |  |  |  |  |  |  |  |  |  |  |  |  |  |  |  |  |  |
| bio13 |  |  |  |  |  |  |  |  |  |  |  |  |  |  |  |  |  |  |
| **bio14** |  |  |  |  |  |  |  |  |  |  |  |  |  |  |  |  |  |  |
| **bio15** |  |  |  |  |  |  |  |  |  |  |  |  |  |  |  |  |  |  |
| bio16 |  |  |  |  |  |  |  |  |  |  |  |  |  |  |  |  |  |  |
| bio17 |  |  |  |  |  |  |  |  |  |  |  |  |  |  |  |  |  |  |
| **bio18** |  |  |  |  |  |  |  |  |  |  |  |  |  |  |  |  |  |  |
| **bio19** |  |  |  |  |  |  |  |  |  |  |  |  |  |  |  |  |  |  |

**Table S10. The contributions of the 11 bioclimatic variables (abbreviation in parentheses) to the Maxent models during ENM analysis for total and geographic populations, respectively, with their permutation importance index indicated in parentheses.** Values corresponding to the three most significant variables are in red.

|  | Total | Europe | Central Asia | Eastern China |
| --- | --- | --- | --- | --- |
| AUC value |  |  |  |  |
| Training data | 0.967 | 0.985 | 0.97 | 0.997 |
| Test data | 0.957 | 0.973 | 0.971 | 0.986 |
| Contribution of each bioclimatic variable |  |  |  |  |
| Mean Monthly Temperature Range (bio2) | 0.079 (0.111) | 0.035 (0.011) | 0.005 (0.035) | 0.072 (0.036) |
| Isothermality (bio3) | **0.368 (0.384)** | **0.19 (0.27)** | **0.319 (0.357)** | **0.177 (0.029)** |
| Max Temperature of Warmest Month (bio5) | 0.056 (0.046) | 0.063 (0.038) | **0.17 (0.043)** | **0.109 (0.16)** |
| Temperature Annual Range (bio7) | **0.124 (0.015)** | **0.256 (0.473)** | 0.007 (0) | 0.097 (0) |
| Mean Temperature of Wettest Quarter (bio8) | 0.033 (0.023) | 0.104 (0.033) | 0.003 (0.078) | 0.001 (0) |
| Mean Temperature of Driest Quarter (bio9) | 0.11 (0.077) | 0.044 (0.023) | 0.109 (0.005) | 0.084 (0.771) |
| Annual Precipitation (bio12) | 0.02 (0.139) | 0.079 (0.078) | 0.035 (0.168) | 0.001 (0) |
| Precipitation of Driest Month (bio14) | 0.011 (0.042) | 0.001 (0.004) | 0.085 (0.006) | 0.031 (0.001) |
| Precipitation Seasonality (bio15) | 0.031 (0.092) | 0.032 (0.039) | 0.008 (0.014) | 0.023 (0.002) |
| Precipitation of Warmest Quarter (bio18) | 0.009 (0.049) | 0.002 (0.012) | **0.151 (0.257)** | **0.404 (0)** |
| Precipitation of Coldest Quarter (bio19) | **0.159 (0.022)** | **0.194 (0.018)** | 0.08 (0.038) | 0.001 (0) |

**Table S11. Markers used in the QTL mapping analysis.**

|  | **Forward primer** |  |  | **Reverse primer** |  | **Type** |
| --- | --- | --- | --- | --- | --- | --- |
| **Name** | **Position** | **sequence** | **Name** | **Position** | **sequence** |  |
| G2832* | Chr1: 622434-622454 | GGATGGATCAGTATGAGATTC | G2833* | Chr1: 623086-623066 | AGTGGCTCACACTAAATCCCA | Indel |
| G2541* | Chr1: 5873112-5873131 | GCGTAATACGTTGCATTAGC | G2542* | Chr1: 5873622-5873641 | CGATTCCAATTATTGGTTCG | Indel |
| G2836* | Chr1: 10666400-10666380 | TGATCTGACGTGATAGTATTC | G2837* | Chr1: 10665629-10665649 | AGTGACTGATAAGCTGGACAA | Indel |
| G2966* | Chr1: 18546485-18646505 | CACTGCCAATGCATGCATAGG | G2967* | Chr1: 18547364-18547344 | GCCTGGACTGGACATTGACAT | SNP |
| G2838* | Chr1: 30187368-30187388 | ATGTCACTTGATTCAAGTCAC | G2839* | Chr1: 30188440-30188420 | TACCTCGGAAGCTGATGGCAT | Indel |
| G2874* | Chr2: 1318138-1318158 | ACTAATGCGGAATCATTACTG | G2875* | Chr2: 1318801-1318781 | ATGCCTTGGTGAATCATACCT | Indel |
| G1956* | Chr2: 3567846-3567865 | CCAGACTCAAAGACCGACTC | G1957* | Chr2: 3569138-3569119 | CACTTCCACAATTATCCGCA | Indel |
| G2576* | Chr2: 5664871-5664891 | CTGAGACTGTAACATTCAGCT | G2577* | Chr2: 5665310-5665290 | ATGCTGTTGATCACTTGGCTG | Indel |
| G2610* | Chr2: 7417920-7417900 | ACTAGTTGTGTGTGCTAGATC | G2611* | Chr2: 7416218-7416238 | GGTTAACTTATGAACCAGTGC | Indel |
| G2608* | Chr2: 8800604-8800624 | AGCAACTCCTAAAGGTGTCCT | G2609* | Chr2: 8800976-8800956 | CAAGCAAACAATGTCGAGAGC | Indel |
| G2660 | Chr2: 9295265-9295284 | GGGAGCTGAATTTGAGACAC | G2661 | Chr2: 9295912-9295893 | TGCTTGTATCTGACCTCTGC | SNP |
| G2753 | Chr2: 9503558-9503535 | TCGCAGTCGGCGATCGAACGACGT | G2754 | Chr2: 9502404-9502427 | CTCTAGTGCATCTCGGTCATTGAT | SNP |
| G2798 | Chr2: 9547395-9547415 | GCACATAGTGCTTATCATGGC | G2799 | Chr2: 9548286-9548266 | CTACCGTGATTCCACTGTATC | SNP |
| G2708 | Chr2: 9580153-9580172 | CACGATTTACTTTCCATTTC | G2709 | Chr2: 9580865-9580846 | TACCGTAGTTAGATACCTTA | SNP |
| G2777* | Chr2: 9615467-9615486 | GCCCGTGAGTCCAAGAAGAG | G2778* | Chr2: 9616302-9616283 | ACCATCAGCCGTCAATGTCC | Indel |
| G2796 | Chr2: 9648092-9648111 | ATGTGTACTTCTTCGTAATGG | G2797 | Chr2: 9648867-9648846 | GCTGGTTGAGATAGACATGTG | SNP |
| G2739 | Chr2: 9678029-9678048 | CTTACCCGATTTCTCACTAG | G2740 | Chr2: 9678751-9678732 | GATAGGTTAGGTTCCTCGTC | SNP |
| G2664 | Chr2: 9726998-9726979 | GAACCCTGAACACAATAACC | G2665 | Chr2: 9726351-9726370 | TCTGAATTGACTCCAAACCG | SNP |
| G2632 | Chr2: 10012074-10012093 | TGAGACAATCACAACTTTCG | G2633 | Chr2: 10011660-10011679 | GAACCACGGGTACGATATTC | SNP |
| G2624 | Chr2: 10538381-10538400 | CTAGTTAACTTCGTAGTTCG | G2625 | Chr2: 10537897-10537916 | TAAACTGTGTAGTAGATCTC | Indel |
| G2626 | Chr2: 10573818-10573837 | CCGATTCTGAATTCTGCTAG | G2627 | Chr2: 10574292-10574311 | ACACGACCCGGATACACAAG | Indel |
| G2612* | Chr2: 11818064-11818083 | GGAACCTAGATTCAGATTCG | G2613* | Chr2: 11818396-11818415 | GCTCAATATTTCGATTGACG | Indel |
| G2578* | Chr2: 14880703-14880683 | ATCTAGAGCCGGACAGCATGC | G2579* | Chr2: 14879951-14879971 | CACAAACCGACATTAACCTTG | Indel |
| G1039* | Chr2: 16094570-16094589 | AATCTGAGGACAAGGGCACA | G1040* | Chr2: 16095178-1609519 | AAACCTGCATTTAAGACCAA | Indel |
| G2840* | Chr3: 1108123-1108103 | ATGGAGTTAGTGTACTAATCT | G2841* | Chr3: 1107638-1107658 | ATCGCAACCCTCGATCCTCTG | Indel |
| G2594* | Chr3: 5585806-5585826 | CACGTATCGTGGATATATCGC | G2595* | Chr3: 5586230-5586210 | CTGTGAACCATTCTCGTACAC | Indel |
| G2842* | Chr3: 9266025-9266005 | GATCTCAATGAGTGCTCAAGT | G2843* | Chr3: 9264582-9264602 | TGTCCATGATGTCAACATGTA | Indel |
| G2955* | Chr3: 15398894-15398914 | TGATGACGGAGAAGATACAGT | G2956* | Chr3: 15399694-15399674 | TGTACACTGTCTTGCACCATG | Indel |
| G2582* | Chr3: 18774600-18774620 | GTGATGCTGCCTTTATCGGCC | G2583* | Chr3: 18775325-18775305 | GACAGCAATAGAACGCTAACA | Indel |
| G2844* | Chr3: 22680238-22680258 | TGACGAGATCAACCGCAACCA | G2845* | Chr3: 22680617-22680597 | GCCATGATAGGCCAAGTTTAG | Indel |
| G2828* | Chr4: 2775372-2775353 | ACATGGAATTGCATCACATG | G2829* | Chr4: 2774754-2774773 | CATTCAAACGTCATTACAAG | Indel |
| G2802* | Chr4: 7103000-7103019 | TACCACCGATATTGCGAATC | G2803* | Chr4: 7103506-7103487 | CGACAGACAAGTTAAGTGCC | Indel |
| G2824* | Chr4: 12268797-12268778 | GTCTGAGCTATGAGCTTAGG | G2825* | Chr4: 12268010-12268029 | TGAATAACCAGAGACAGAGG | Indel |
| G2826* | Chr4: 12341834-12341815 | GCCGCAGAGTGATTGTTTAG | G2827* | Chr4: 12340978-12340997 | TTTCAGAGCAGACCCATGTG | Indel |
| G2586* | Chr4: 13486791-13486811 | AGCCTGTGAGGGTATTATTGT | G2587* | Chr4: 13487268-13487248 | AGTGTCTCTCATGCGTCAAAT | Indel |
| G2971* | Chr4: 18081953-18081935 | CACCTTTTGGTTCTGTGCC | G2972* | Chr4: 18081424-18081442 | CTCCTGAGGTGCGTGTATG | SNP |
| G2640* | Chr5: 2639080-2639098 | TCTTCAGATTCCGTTACCT | G2641* | Chr5: 2639534-2639516 | GTTCTACCTTCGCTGAGAC | Indel |
| G2864* | Chr5: 4582999-4582980 | CTGTCTTAACCCATAATGTC | G2865* | Chr5: 4582207-4582225 | TGCTCTACGAATACGAAAC | Indel |
| G2650* | Chr5: 5473426-5473444 | CTTTCCTTCACTGGTCTAA | G2651* | Chr5: 5473812-5473794 | AGGGATAAATAAGGGAGTC | Indel |
| G2910* | Chr5: 13945963-13945944 | CTCAGCCGTAAGTAAATAAG | G2911* | Chr5: 13945208-13945226 | GAAGCAGAAAGTCTCCCAC | Indel |
| G2600* | Chr5: 21976399-21976379 | CATCTCTGGCTAGACTAATAG | G2601* | Chr5: 21975672-21975692 | ACTAGATCCCACACACGATTG | Indel |
| G2912* | Chr5: 24704084-24704066 | ACAAATGATAAGGGAAGTC | G2913* | Chr5: 24703267-24703285 | ACGTCGGTTAAGCAAATAC | Indel |

“*” indicates markers used for R/qtl analysis.
