# Supplementary material for: Adaptation of Arabidopsis thaliana to the Yangtze River basin
Source: Genome Biol. 2017 Dec 28;18:239. doi: 10.1186/s13059-017-1378-9 (PMC5745794; doi:10.1186/s13059-017-1378-9)
Supplement: Supplementary file 2 — PCA analysis of ecological differentiation among strains of A. thaliana based on 19 environmental variables using two discriminant principal components (PC) based on 291 geo-referenced and non-overlapped occurrence records of A. thaliana (Additional file 5: Table S5). Bio4 and bio12 are the most significant factors differentiating PC2 and PC1, respectively. All the pairwise comparisons of bio4 or bio12 among the three regions are significant (P < 0.001). Figure S2. Sequence variation of the 118 strains sequenced in this study. Intergenic represents intergenic region; gene represents genic region from ATG to TGA/TAA/TAG, including introns; 5’ UTR represents the untranslated region before start codon ATG; CDS represents coding sequence region; 3’ UTR refers the untranslated region after stop codon (TGA/TAA/TAG); total represents whole genome in total. Figure S3. Phylogenetic tree of all the 221 A. thaliana strains with outgroups. Numbers nearby a branch indicate the bootstrap value > 50% with 100 replicates. Strains from different regions were color-coded, pink: European strains, blue: central Asia strains, yellow: eastern Asia strains, green: others indicate strains from USA, Japan, and New Zealand, most probably reflects recent introduction, given A. thaliana originated in Europe. Figure S4. Saturation analysis of the 30 times random samplings of the Yangtze River population (popY) based on the recovery of the number of total SNPs. Figure S5. Genetic variation among different populations. Figure S6. Different models of demographic history between the two populations of A. thaliana. Model 4 is the best fit model; see Table S4 for the detailed demographic parameters for each model. Figure S7. Selection scans of genes under positive selection based on LD-based method (OmegaPlus). The dashed red line indicates the threshold of 0.01% based on simulation data sets. Figure S8. Over-representation (FDR < 0.01) of GO annotation categories in gene sets under selection. (DOCX [file 13059_2017_1378_MOESM2_ESM.docx]

**
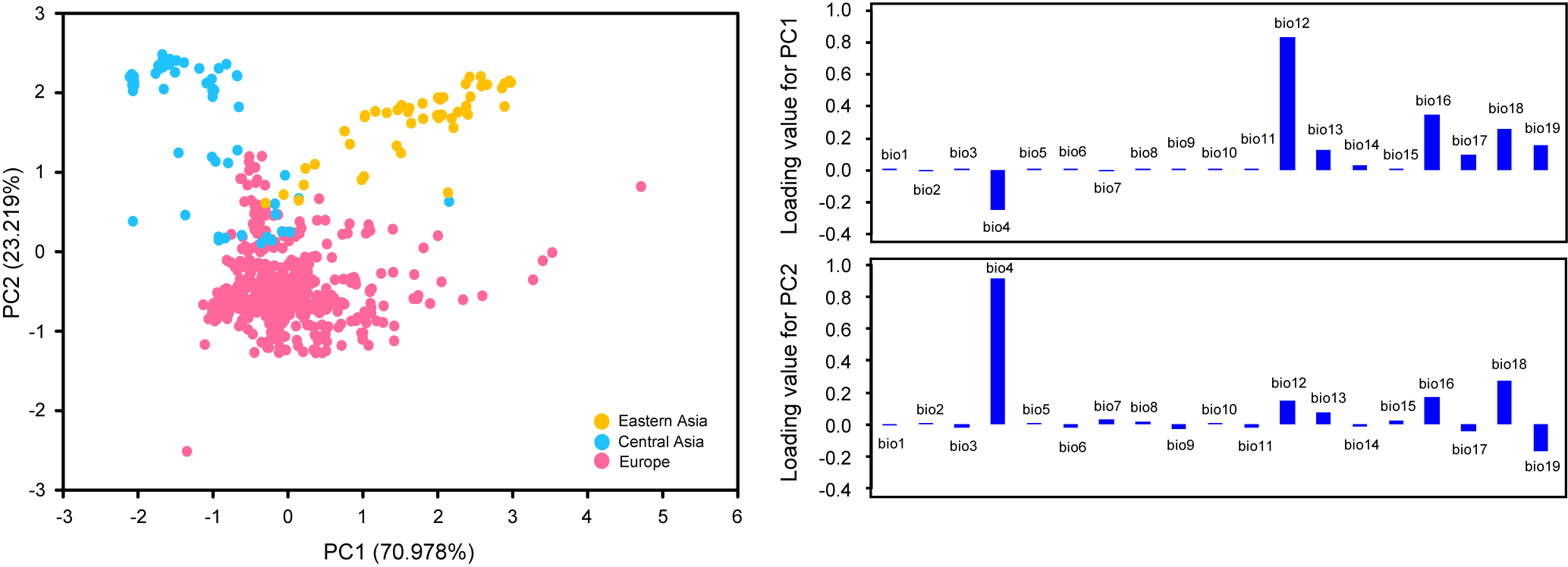
**

**Figure S1. PCA analysis of ecological differentiation among strains of *A. thaliana* based on 19 environmental variables using two discriminant principal components (PC) based on 291 geo-referenced and non-overlapped occurrence records of *A. thaliana* (Additional file 5: Table S5).** Bio4 and bio12 are the most significant factors differentiating PC2 and PC1, respectively. All the pairwise comparisons of bio4 or bio12 among the three regions are significant (*P* < 0.001).


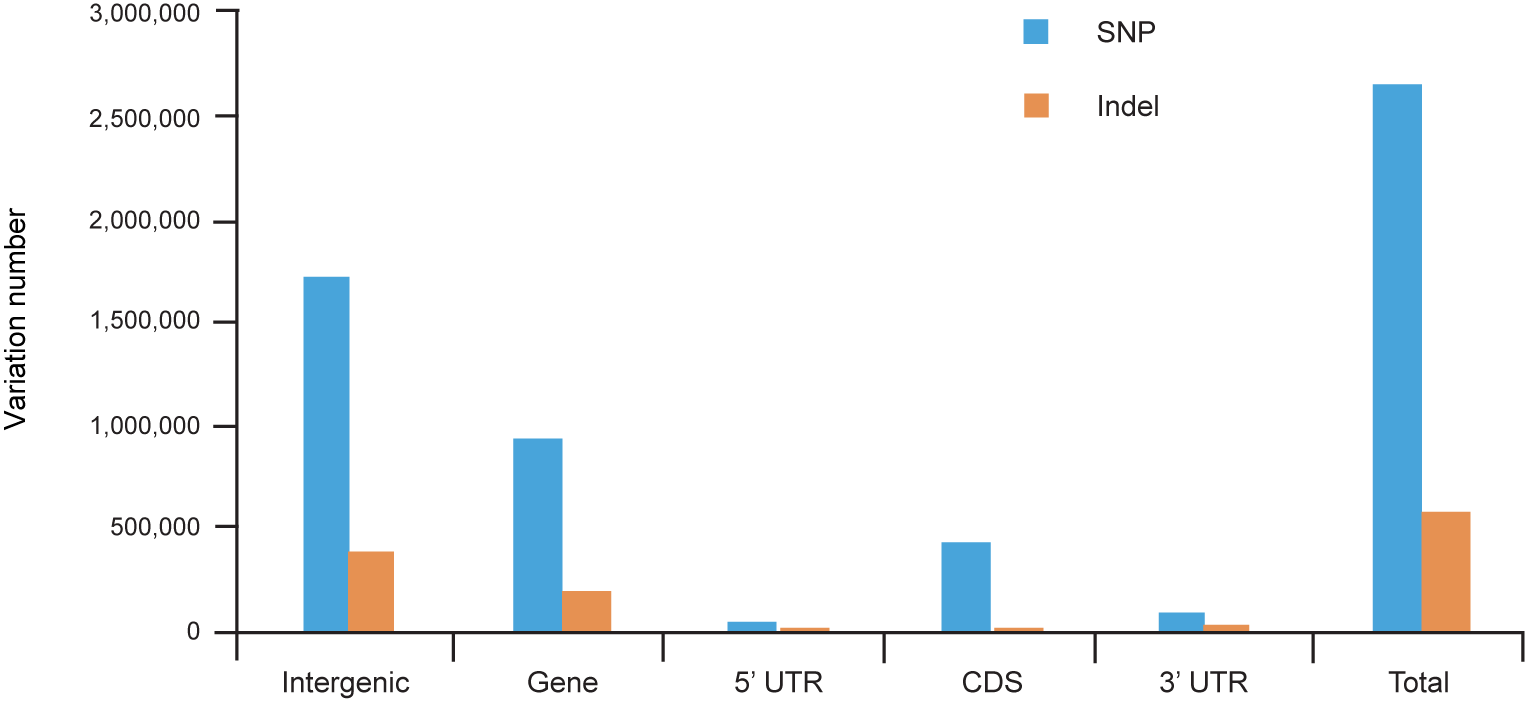


**Figure S2.** **Sequence variation of the 118 strains sequenced in this study.** Intergenic represents intergenic region; gene represents genic region from ATG to TGA/TAA/TAG, including introns; 5’ UTR represents the untranslated region before start codon ATG; CDS represents coding sequence region; 3’ UTR refers the untranslated region after stop codon (TGA/TAA/TAG); total represents whole-genome in total.

**
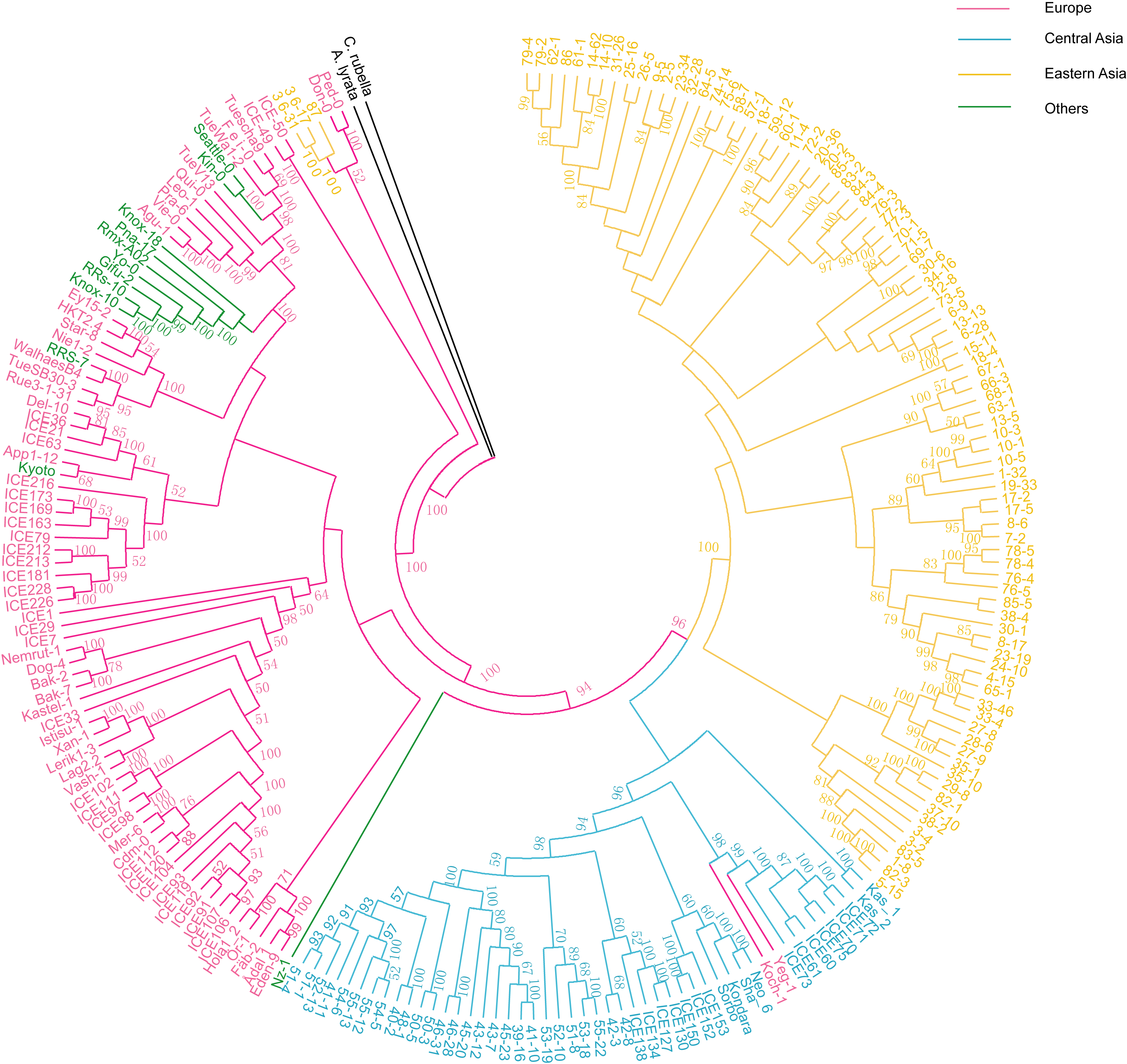
**

**Figure S3. Phylogenetic tree of all the 221 *A. thaliana* strains with outgroups.** Numbers nearby a branch indicate the bootstrap value above 50% with 100 replicates. Strains from different regions were color-coded, pink: European strains, blue: central Asia strains, yellow: eastern Asia strains, green: others indicates strains from USA, Japan, and New Zealand, most probably reflects recent introduction, given *A. thaliana* originated in Europe.


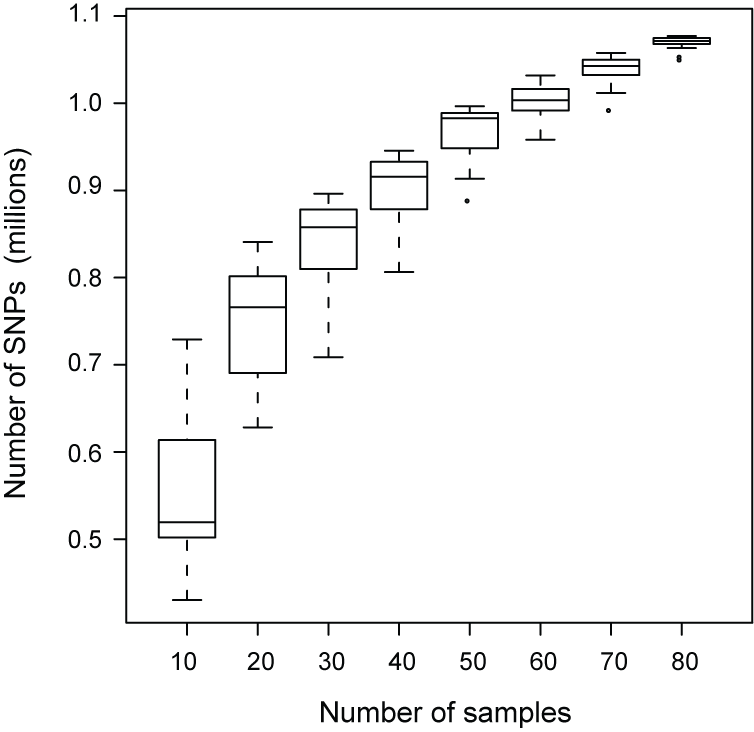


**Figure S4. Saturation analysis of the 30 times random samplings of the Yangtze River population (popY) based on the recovery of the number of total SNPs.**

**
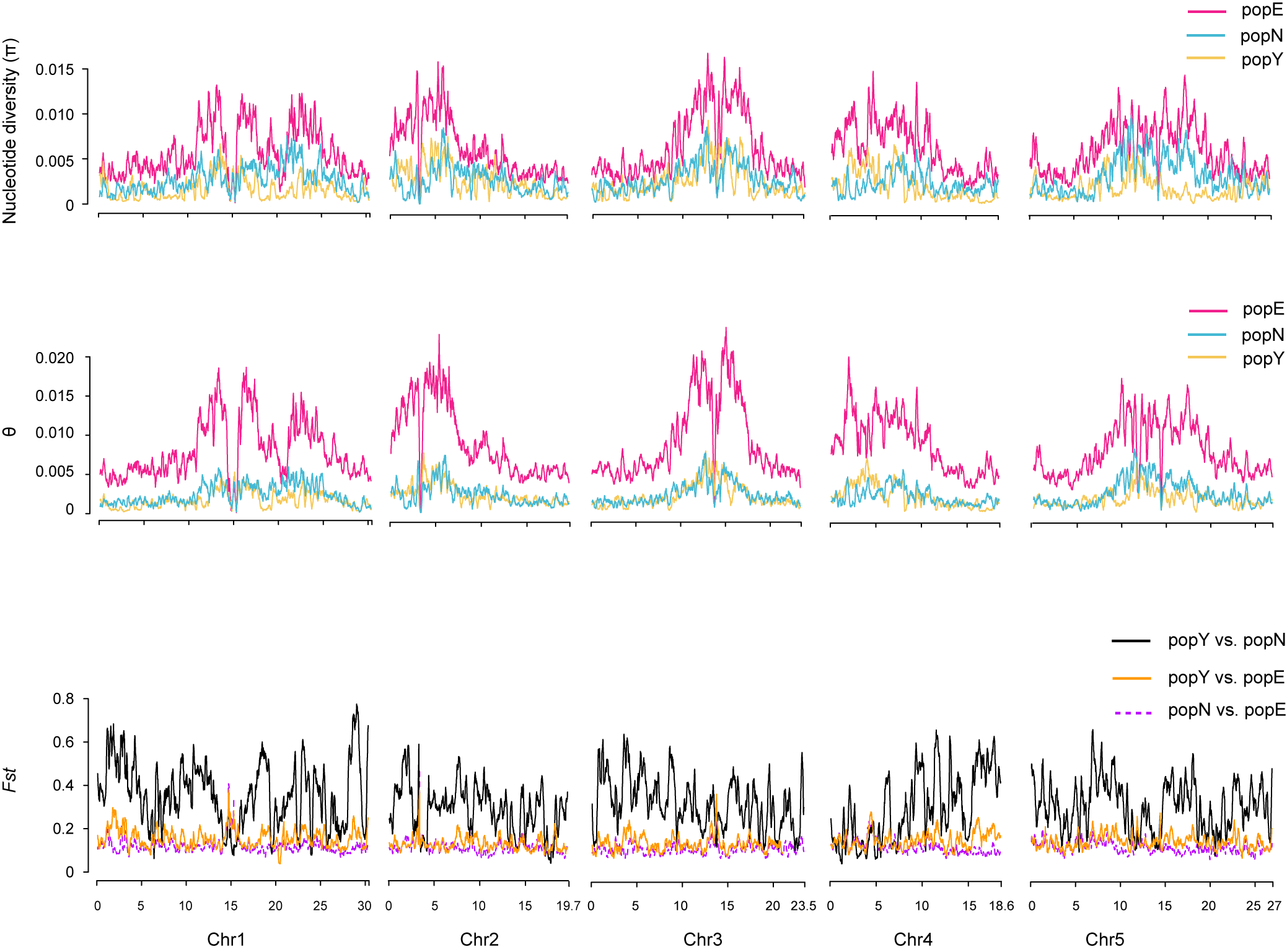
**

**Figure S5. Genetic variation among different populations.**


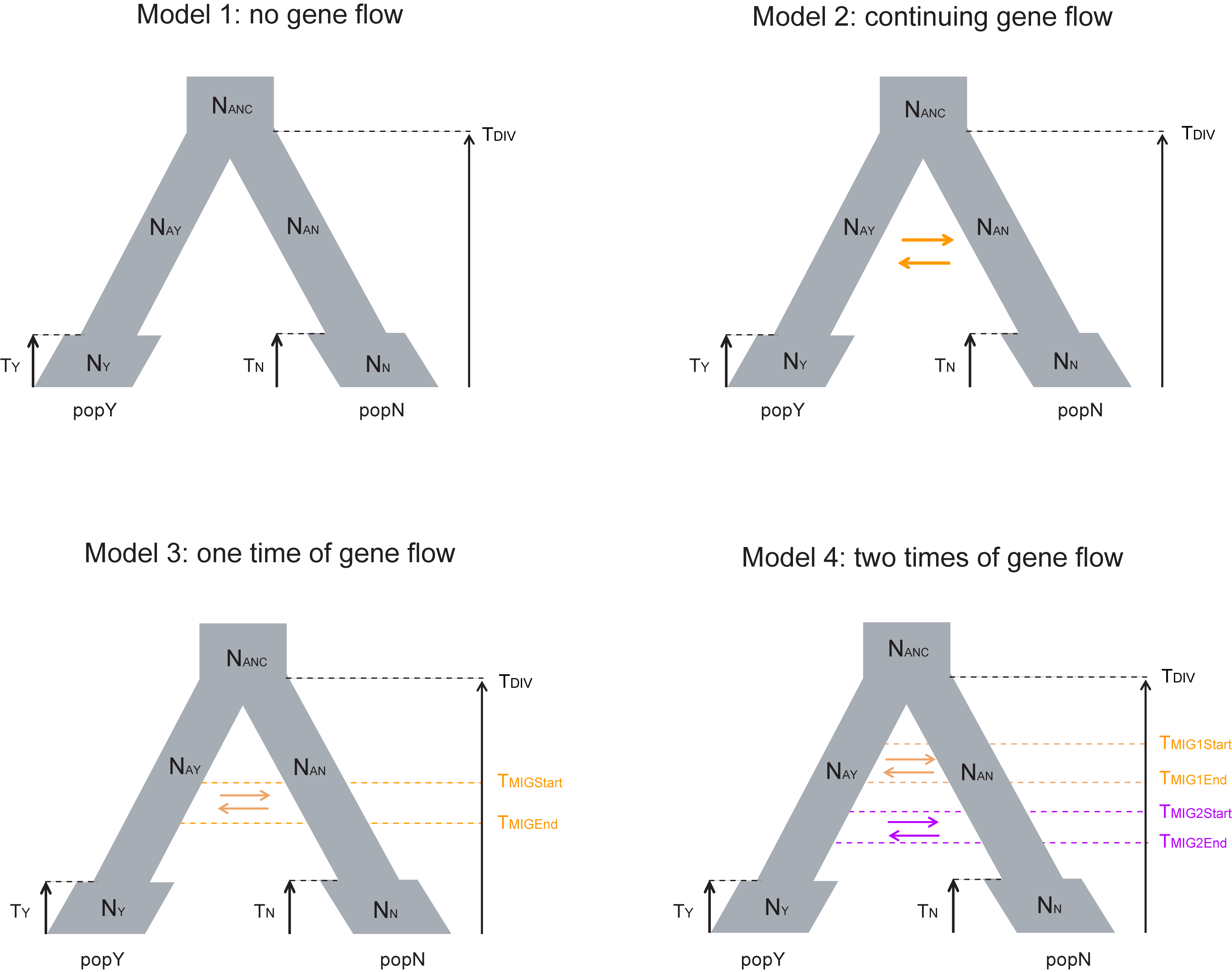


**Figure S6. Different models of demographic history between the two populations of *A. thaliana*. Model 4 is the best fit model, and see Table S4 for the detailed demographic parameters for each model.**


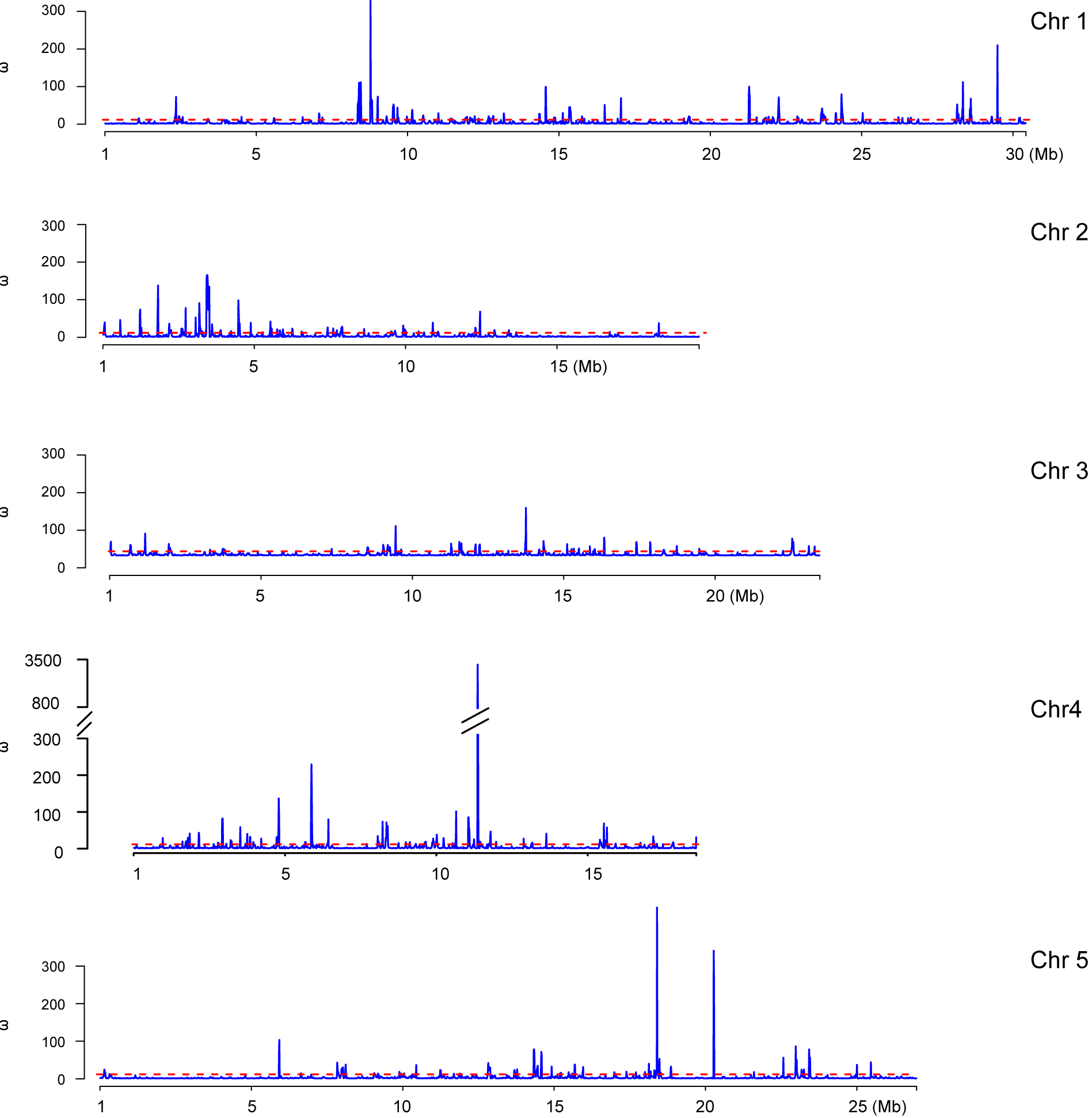


**Figure S7. Selection scans of genes under positive selection based on LD-based method (OmegaPlus). The dashed red line indicates the threshold of 0.01% based on simulation data sets.**

**
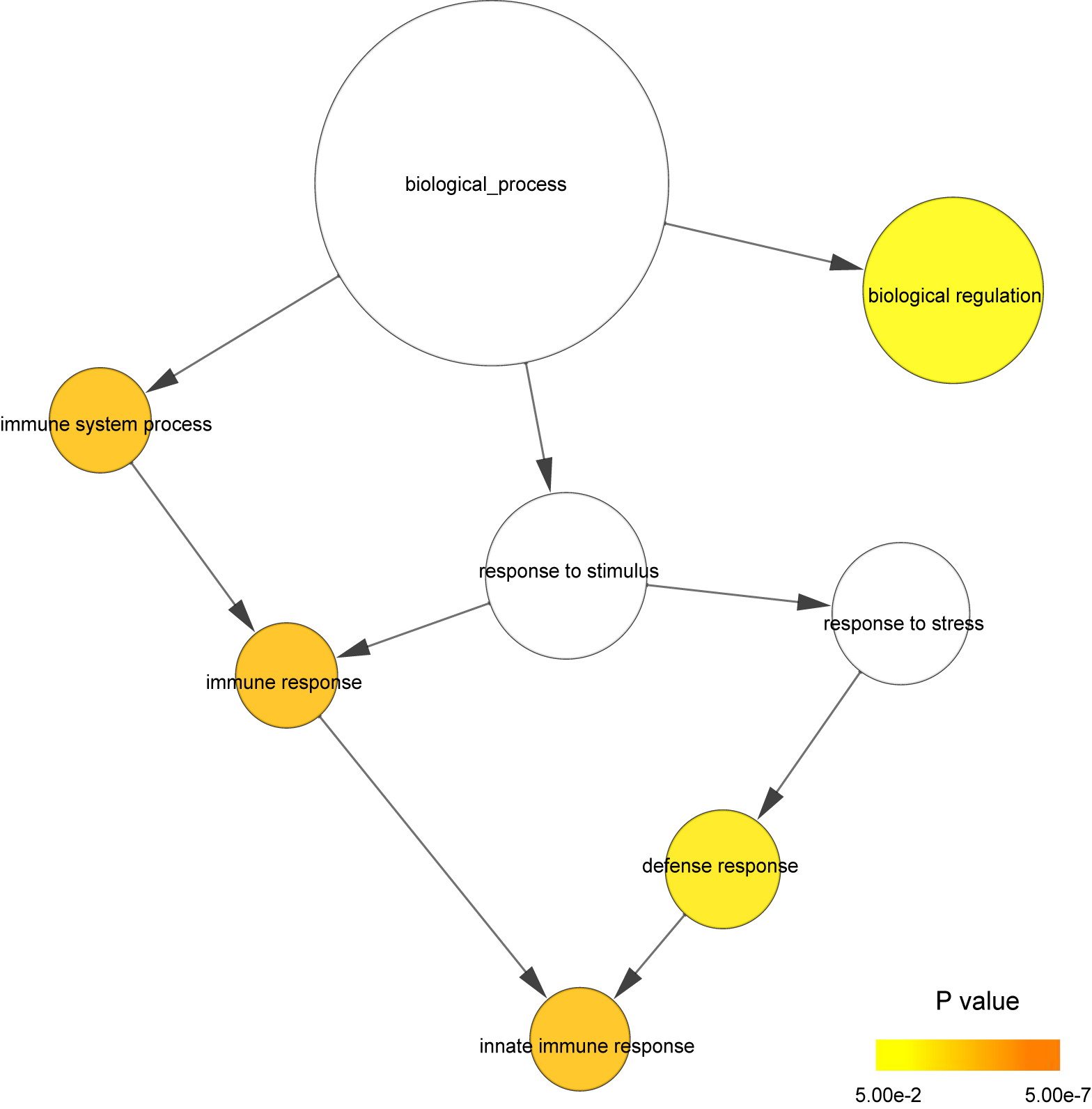
**

**Figure S8. Overrepresentation (FDR < 0.01) of GO annotation categories in gene sets under selection.**
